# No Right to Trifle with Human Life

**Published:** 1886-05

**Authors:** 


### No Right to Trifle With Human Life.

Human life is too precious to be thrown away in mere experimentation, and the man who inoculates himself with the virus of an unknown disease, or who doses himself with a new drug in order to observe its toxic effect, without having previously ascertained its strength, is a fool, albeit a noble one. We have the lower animals to experiment upon, and we have no right to trifle with human life, whether it be our own or that of our neighbor. —*Medical Record.*

